# El Niño and eye health: ophthalmic manifestations of changes in climate

**DOI:** 10.1038/s41433-023-02907-z

**Published:** 2024-01-04

**Authors:** Ethan Waisberg, Joshua Ong, Andrew G. Lee

**Affiliations:** 1 Department of Ophthalmology, University of Cambridge, Cambridge, UK; 2 Michigan Medicine, University of Michigan, Ann Arbor, MI USA; 3 Center for Space Medicine, Baylor College of Medicine, Houston, TX USA; 4 Department of Ophthalmology, Blanton Eye Institute, Houston Methodist Hospital, Houston, TX USA; 5 The Houston Methodist Research Institute, Houston Methodist Hospital, Houston, TX USA; 6 Departments of Ophthalmology, Neurology, and Neurosurgery, Weill Cornell Medicine, New York, NY USA; 7 Department of Ophthalmology, University of Texas Medical Branch, Galveston, TX USA; 8 University of Texas MD Anderson Cancer Center, Houston, TX USA; 9 Texas A&M College of Medicine, Bryan, TX USA; 10 Department of Ophthalmology, The University of Iowa Hospitals and Clinics, Iowa City, IA USA

**Keywords:** Risk factors, Public health

El Niño is a climate phenomenon which results in the warming of sea temperatures in the Pacific Ocean, which causes shifts in weather patterns worldwide. El Niño conditions have recently developed for the first time in seven years according to the World Meteorological Organization [1]. This can lead to more extreme conditions such as extreme heat, increased floods and droughts. Climate change has recently been implicated in the increased frequency and severity of El Niño events [2]. While El Niño events are known to primarily affect ecosystems and weather patterns, new research suggests that it can have significant impacts on human health [3]. Rising temperatures are associated with an increased cardiovascular risk [4], while smoke from wildfires and increased particulate matter and atmospheric dust can lead to respiratory system damage [5]. While our group typically focuses on ocular health of astronauts in the austere environment of space [6–8], we explore in this paper how El Niño events and alterations in climate can potentially impact eye health on Earth.

## Vector-borne diseases

Higher temperatures and increased levels of rainfall can produce favorable conditions for mosquitoes, which are disease-carrying vectors. Warmer climates also lead to an extended disease transmission season while also increasing the biting behaviour of mosquitoes, further increasing the risks of these disease-carrying vectors [9]. Mosquitoes can carry organisms that cause significant vision loss and ocular pathology, including: dengue viruses, West Nile, chikungunya, malarial parasites, encephalitis viruses etc. [10]. Eye care providers in regions that have become warmer, may also be may not be as familiar with ocular findings of mosquito-transmitted diseases and this may result in delays in treatment, and lead to further decrements in visual function.

## Drought

El Niño events can typically cause higher levels of rainfall in the southern United States, central Asia, and southern South America, while causing severe droughts in regions such as Central America, southern Asia, Indonesia and Australia [1]. Regions experiencing drought can affect the hydration status of the individuals living in that region. Alterations in the systemic hydration status of an individual can potentially affect the development of a variety non-ophthalmic and ophthalmic diseases. The eye may particularly be vulnerable to the effects of dehydration due to its high water content [11]. In a systematic review by Sherwin et al. [11], dehydration was found to potentially be associated with dry eye syndrome, retinal vascular disease, refractive changes, and cataract. Further research is required to examine the effects of chronic and acute dehydration on the eye, particularly in individuals with various eye diseases.

## Increased levels of precipitation

Increased levels of precipitation can result in flash flooding, which could lead to water contamination. Developing countries are particularly at risk to this contamination due to poorer drainage system infrastructure. Pathogens linked to regional flooding, and water contamination include toxoplasmosis which can cause retinochoroiditis [12], or acanthamoeba which can cause a keratitis [13].

## Ultraviolet radiation exposure

Alterations in atmospheric conditions and cloud cover as a result of El Niño events can potentially lead to higher levels of ultraviolet radiation (UV) levels. Ultraviolet radiation has been linked to a variety of ophthalmic conditions, including: pterygium, photokeratitis, and eyelid malignancies such as squamous cell carcinoma and basal cell carcinoma [14]. Ultraviolet radiation exposure has also been implicated in the development of cataracts [15, 16] and choroidal folds [17, 18].

## Elevated temperatures

Elevated temperatures can also potentially lead to increased retinal pathology. A nationwide, 11-year study in Taiwan by Lin et al. [19]. found a significant increase in retinal detachments among higher ambient temperatures. Another study also found a significant increase in retinal detachments in Quebec following a heat wave [20].

Although the relationship between eye health, climate, and El Niño events is a multifaceted and highly complex topic, climate variability can potentially have significant impacts on ocular health. Further research, and vision screening [21, 22] is required to examine the impacts of alterations in climate on eye health. This work will be essential to inform public health preparedness and help to mitigate the impacts of climate on ocular health.
